# Identification and preliminary clinical validation of type 2 diabetes signature genes through machine learning analysis of scRNA-seq data

**DOI:** 10.3389/fmed.2026.1801737

**Published:** 2026-05-12

**Authors:** Fang Tang, Xin Zuo, Weiyan Wang, Rui Sun, Heng Cheng, Weihua Wu

**Affiliations:** Department of Endocrinology, Shenzhen Third People’s Hospital, The Second Affiliated Hospital of Southern University of Science and Technology, Shenzhen, Guangdong, China

**Keywords:** clinical validation, machine learning, Signature genes, single-cell RNA sequencing, type 2 diabetes

## Abstract

**Objective:**

Type 2 diabetes (T2DM) is a highly prevalent metabolic disorder with substantial molecular heterogeneity, and traditional bulk transcriptomic approaches often fail to capture cell-specific changes critical to disease pathogenesis. This study aims to identify and validate key signature genes for T2DM by integrating single-cell RNA sequencing (scRNA-seq) with machine learning, providing new insights into disease mechanisms and potential biomarkers.

**Methods:**

We analyzed scRNA-seq data to characterize cellular heterogeneity across 10 distinct cell types. Differential expression analysis identified 455 candidate genes, which were refined using LASSO regression. The diagnostic potential of identified genes was evaluated using ROC curve analysis on an independent dataset. Functional enrichment and cell communication analyses were performed to elucidate biological processes and intercellular signaling networks. Finally, expression changes of the candidate genes were validated in peripheral blood from a separate clinical cohort (15 T2DM patients, 20 controls) using qRT-PCR.

**Results:**

Four core genes (PNLIP, BUB1, CTSB, NAMPT) were identified as candidate signature genes. ROC analysis showed AUC values of 0.819, 0.931, 0.882, and 0.694, respectively, suggesting promising but variable diagnostic accuracy. Enrichment analyses indicated these genes participate in processes including extracellular matrix remodeling, digestion/absorption, and signal transduction. Cell communication analysis suggested a potential central role of Alpha and Beta cells in diabetic signaling networks, with the MK and SPP1 pathways showing complementary expression patterns. In addition, qRT-PCR confirmed significantly up-regulated expression of PNLIP, BUB1, and CTSB along with down-regulated NAMPT in T2DM patients, supporting their potential as circulating candidate biomarkers.

**Conclusion:**

This study integrates machine learning with scRNA-seq to identify PNLIP, BUB1, CTSB, and NAMPT as potential T2DM signature genes. These findings offer candidate diagnostic biomarkers and provide preliminary mechanistic insights into disease-associated pathways.

## 1 Introduction

Diabetes is a highly prevalent metabolic disease, with T2DM accounting for approximately 90% of cases and standing as a major threat to human health (1). Although traditional studies have revealed part of the pathogenesis at the genetic and transcriptional levels, T2DM exhibits significant heterogeneity in molecular mechanisms and clinical outcomes, stemming from complex interactions between genetic and environmental factors (2, 3). The emergence of single-cell sequencing technologies offers a new perspective, enabling comprehensive detection of genomic, transcriptomic, and epigenomic features at single-cell resolution, thereby helping to elucidate cellular heterogeneity and underlying molecular pathways in disease (4, 5). Therefore, integrating single-cell data to screen for signature genes is crucial for deepening the understanding of T2DM pathogenesis. Recent studies have successfully employed integrated bioinformatics to identify shared molecular signatures between T2DM and other diseases, such as myocardial infarction (6), clear-cell renal cell carcinoma (7), kidney cancer (8), and colorectal cancer (9), demonstrating the power of transcriptomics-based discovery. However, systematic screening of T2DM-specific signature genes using single-cell resolution remains limited.

As a high-throughput technology, scRNA-seq has been widely used in diabetes research, particularly in mapping pancreatic islet cell atlases and identifying features of cellular heterogeneity. For instance, by performing scRNA-seq on islet cells from T2DM patients and normal controls, researchers have been able to identify DEGs in macrophages, revealing potential causal genes for β-cell dysfunction (10, 11). This technology not only identifies cell subpopulations but can also be applied to peripheral blood mononuclear cell analysis, systematically screening for key genes associated with diabetes through T-cell receptor repertoires and gene expression profiles (12, 13). Simultaneously, single-cell sequencing overcomes the limitations of traditional bulk RNA sequencing, allowing precise resolution of cell-specific changes in tissues such as the retina and kidney, thereby providing a high-resolution data foundation for discovering signature genes (14).

The screening of signature genes relies on the integration of various computational and experimental methods. Machine learning algorithms (such as LASSO regression and SVM-RFE) are often used to identify key genes from single-cell data, and their diagnostic value is validated through protein-protein interaction networks and ROC curve analysis (15–17). Immune cell infiltration analysis and microenvironment assessment further reveal the correlation between signature genes and immune responses; for example, in NAFLD models, single-cell data have been used to validate the spatial expression patterns of genes in different cell clusters (such as hepatocytes) (18, 19). Furthermore, wet-lab experimental methods like qRT-PCR are used to cross-validate expression changes of candidate genes in cell models, ensuring the reliability of screening results (20, 21).

This study aims to integrate single-cell transcriptomic data with machine learning algorithms to systematically screen for signature genes of T2DM and validate them using independent cohorts. By deeply analyzing cellular heterogeneity under diabetic conditions, identifying key cell subpopulations and their marker genes, and combining LASSO models to screen for core genes with diagnostic potential, expression changes will be validated in clinical samples via qRT-PCR. This work aims to provide a reliable basis for understanding the molecular mechanisms of T2DM and developing novel biomarkers.

## 2 Materials and methods

### 2.1 Data download and analysis

scRNA-seq dataset GSE221156 from Homo sapiens was downloaded from the Gene Expression Omnibus (GEO) database (accessed on 30 October 2025).^1^ This dataset comprised samples from 7 T2DM patients, 17 non-diabetic individuals, and 14 prediabetic individuals. Additionally, the T2DM microarray dataset GSE29221 was obtained from the GEO database (accessed on 30 October 2025), containing skeletal muscle biopsy samples from three male T2DM patients and three male non-diabetic controls, which was used to validate the signature genes derived from pancreatic islets (22). Although skeletal muscle is not pancreatic tissue, as a major insulin-sensitive organ, it can reflect the systemic metabolic dysregulation of T2DM.

Single-cell data processing was performed using Seurat 4.0. Quality control removed cells with nFeature_RNA < 200 or > 5,000, and with mitochondrial gene percentage > 10%. Data were normalized using NormalizeData (LogNormalize, scale.factor = 10,000). Batch effects across samples from different diabetic statuses were corrected using the Harmony algorithm. Cell types were automatically annotated using the SingleR package. Cell-type-specific genes were identified using the FindAllMarkers function, with significance thresholds set at an adjusted *p*-value < 0.05 and |logFC| > 2. For each of the 19 subclusters, the top 4 marker genes meeting these thresholds were recorded (Supplementary Table 1). To compare cell type proportions across diabetic statuses, the percentage of each annotated cell type within each sample group was calculated.

### 2.2 Identification of DEGs

The scRNA-seq dataset GSE221156 was retrieved from the GEO database using the “GEOquery” package (v2.70.0). Differential expression analysis was performed using the “limma” package (v3.58.1). For enhanced data visualization, volcano plots were generated with the “ggplot2” package (v3.5.0)^2^ to display the distribution of gene expression differences.

### 2.3 LASSO regression analysis

The LASSO-Cox proportional hazards model, implemented via the R package glmnet),^3^ was applied to integrate disease status and gene expression data from the GSE221156 dataset. To ensure reproducibility, the random seed was set to 666. A 5-fold cross-validation was employed to determine the optimal penalty parameter (lambda.1se = 0.093). LASSO regression can automatically address multicollinearity among genes by shrinking the coefficients of redundant genes, making it suitable for high-dimensional single-cell RNA-seq data. A Venn diagram was used to visualize the overlap between genes selected by LASSO regression and the previously identified DEGs. The candidate genes derived from this analysis were subsequently validated using the GSE29221 dataset as an independent validation set. A 5-fold cross-validation was employed to determine the optimal model.

### 2.4 Enrichment analysis

Following candidate gene selection, Gene Ontology (GO) functional enrichment analysis and Kyoto Encyclopedia of Genes and Genomes (KEGG) pathway enrichment analysis were performed using the clusterProfiler package. GO analysis covered three domains: Biological Process (BP), Cellular Component (CC), and Molecular Function (MF). KEGG analysis was used to explore metabolic and signaling pathways associated with the candidate genes. Significantly enriched GO terms and KEGG pathways (*p* < 0.05, FDR < 0.05) were visualized using bubble plots and bar charts.

### 2.5 Cell-cell communication analysis

Cell-cell communication patterns were systematically assessed using the CellChat tool to quantitatively decipher intercellular signaling networks. Standardized data were input into the CellChat platform, and a comprehensive analysis of cell-cell communication within the dataset was performed based on the CellChatDB database (accessed on 6 November 2025).^4^ This analysis quantified interaction strengths and network features between different cell types, providing insights into potential mechanisms of cellular synergy.

### 2.6 Study subjects and clinical procedures

This study is a prospective controlled study conducted between March 2024 and March 2025, enrolling subjects from the Department of Endocrinology of Shenzhen Third People’s Hospital. The participants were divided into two groups: 15 patients with T2DM and 20 age- and gender-matched healthy volunteers, all of whom were over 18 years of age. Patient screening strictly followed international diabetes diagnosis and treatment guidelines and relevant diagnostic criteria, and basic demographic information was collected through the hospital registration system. Exclusion criteria included: diabetic neuropathy, type 1 diabetes (T1DM), secondary diabetes, other acute or chronic metabolic diseases, inflammatory diseases, endocrine system diseases, autoimmune diseases, infectious diseases, malignant tumors, major organ failure, pregnancy, or patients undergoing dialysis therapy. The study protocol was approved by the Ethics Committee of Shenzhen Third People’s Hospital (Approval No.: 2024-019), and all participants signed written informed consent prior to enrollment. Fasting venous blood samples were collected between 8:00 and 8:30 in the morning. Among them, 5 mL of whole blood was collected in EDTA anticoagulant tubes. Samples with visible hemolysis (assessed by visual inspection of plasma color) were excluded to avoid false elevation of RNA concentration and interference with qRT-PCR results.

### 2.7 qRT-PCR analysis

RNA extraction was performed using the BioTeke Human Peripheral Blood RNA Extraction Kit (Model: RP4001) on serum isolated from whole blood. The average RNA yield from 5 mL whole blood-derived serum was 5–10 ng/μL (30 μL elution volume), with A260/280 ratios of 1.8–2.0, indicating acceptable purity for downstream analysis. Due to the fragmented nature of serum cell-free RNA, RNA integrity was assessed by the consistent amplification of GAPDH (Cq 24–28) and single peaks in melt curves across all samples. RNA concentration and purity were measured using a Nano 600 spectrophotometer (Jiapeng Technology, Shanghai, China). Subsequently, RNA was reverse transcribed into cDNA using the HiScript III 1st Strand cDNA Synthesis Kit (R312, Beyotime). qRT-PCR was performed on a CFX96 Touch Real-Time PCR Detection System (Bio-Rad, United States) using Universal SYBR qPCR Master Mix (MQ101, Beyotime). Relative expression levels of genes were calculated using the 2^−ΔΔ^*^Cq^* method, with GAPDH as the internal reference gene. The primer sequences are as follows: PNLIP forward 5′-CACTGCTGCTGGGAGCAGTA-3′, reverse 5′-CCAAGGCAATATATGG AGGG-3′; BUB1 forward 5′-GTGGTGGAGACATCCCATGA-3′, reverse 5′-TTTC CATGTTCACTGGTGTC-3′; CTSB forward 5′-CCTGGGTGG GCCCAAGCCAC-3′, reverse 5′-GCCGACACCTCCACGCTG AC-3′; NAMPT forward 5′-GAGACTGC TGGCATAGGAGC-3′, reverse 5′-CTTCACCCCATATTTTCTCAC-3′; and GAPDH forward 5′-TGGAGAAACCTGCCAAGTATG-3′, reverse 5′-TGGAAGAATGGGA GTTGCT-3′.

### 2.8 Statistical analysis

All data computations and statistical analyses were performed using R software (v4.1.2). For comparisons between two groups of continuous variables, the Mann-Whitney U test (Wilcoxon rank-sum test) was used for non-normally distributed data. Unless otherwise specified, correlation analysis was performed using Spearman’s rank correlation via R’s cor function, and AUC (Area Under the Curve) calculations were implemented using the pROC package.^5^ This statistical strategy integrated non-parametric tests, correlation assessment, and ROC curve analysis to ensure robust handling of data with varying distributions and verification of result reliability.

## 3 Results

### 3.1 Single-cell subpopulation identification and analysis

**FIGURE 1:**
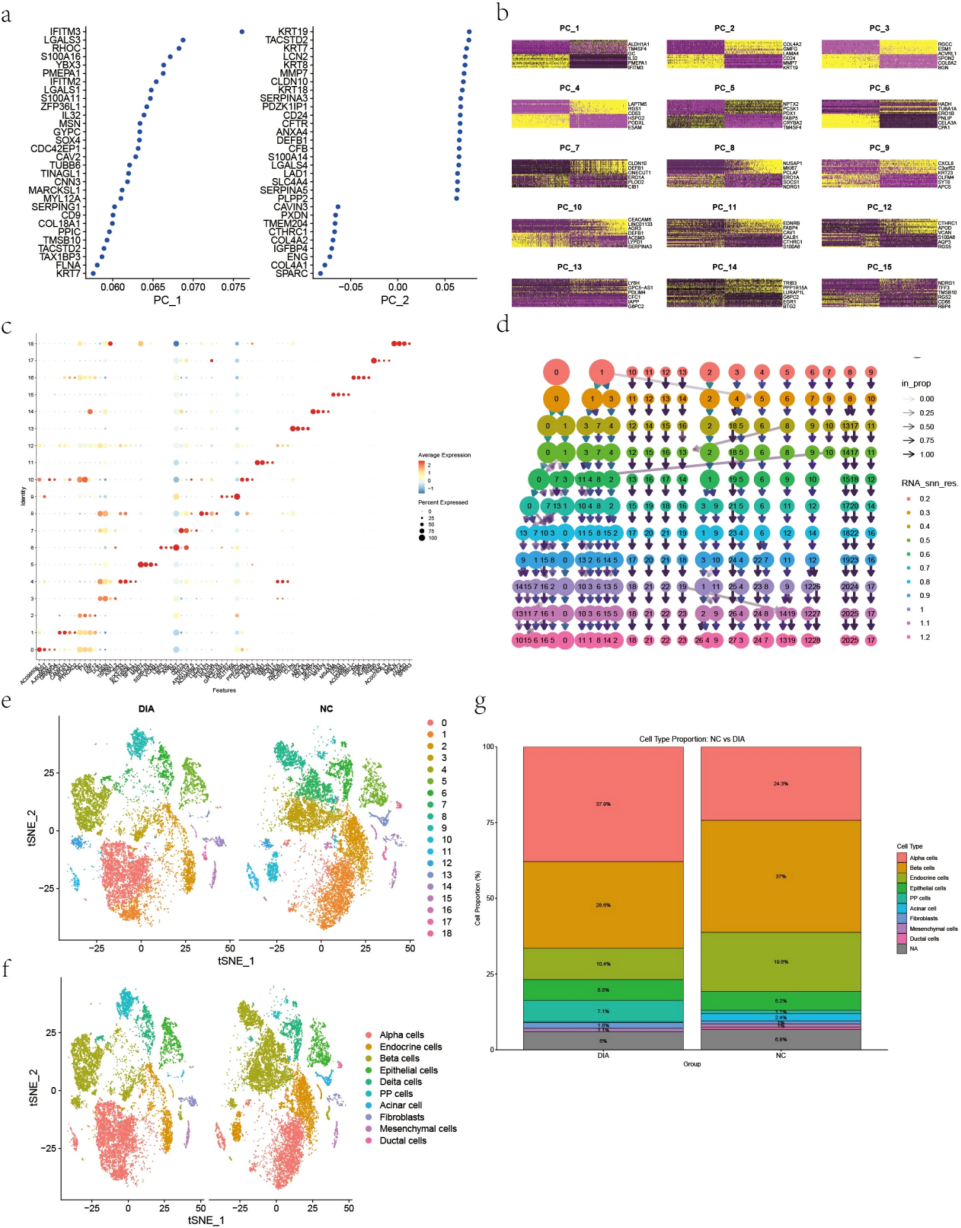
Single-cell data analysis based on the GSE221156 dataset, showing dimensionality reduction, clustering, and specific marker characteristics of different cell types. **(a)** Principal component analysis using the elbow method to identify significant principal components. **(b)** Heatmap of the top 15 principal components, showing the overall structure of gene expression. **(c)** The top four marker genes by expression level in each cell subpopulation, showing significant upregulation in their respective clusters (*p* < 0.05). **(d)** t-SNE visualization of the 19 cell subpopulations obtained at a resolution of 0.5. **(e)** Automated annotation results for the 19 cell subpopulations. **(f)** Manual annotation of subpopulations based on known cell type marker genes, identifying 10 cell types. **(g)** Cell type proportion comparison across T2DM and non-diabetic (ND) groups.

This study was based on the GSE221156 dataset and performed a systematic analysis of scRNA-seq data using the Seurat R package (v4.0). After rigorous quality control, including the removal of low-quality cells and genes, normalization, and highly variable gene selection, 15 Principal Components (PCs) were extracted for subsequent analysis. Principal Component Analysis revealed a clear hierarchical structure in the gene expression patterns contributed by each PC (Figure 1a). A PC heatmap further illustrated the distribution patterns of different genes across multiple principal components (Figure 1b). By adjusting the RNA_snn_res. parameter (from 0.2 to 1.2), the granularity of cell clustering was controlled, allowing the number of clusters to be adjusted from few to many (0–28 clusters). Ultimately, a resolution of 0.5 was selected, partitioning the cells into 19 distinct subpopulations (Figure 1d).

Furthermore, the top four genes with the highest expression levels in each subpopulation were identified (Figure 1c). These genes were expressed significantly higher in their respective cell types compared to others (*p* < 0.05), suggesting their potential as cell type-specific markers. Subsequently, systematic annotation of the 19 subpopulations was performed by integrating automated annotation via SingleR and known cell taxonomy markers, ultimately identifying ten cell types, including alpha cells, beta cells, macrophages, etc. (Figure 1f). t-SNE dimensionality reduction visualization clearly displayed the distribution of different cell subpopulations in two-dimensional space (Figure 1e), establishing a multi-level analytical framework from gene expression to cell typing. We further compared the proportions of the 10 annotated cell types across T2DM and ND. As shown in Figure 1g, the relative abundance of alpha and beta cells differed significantly among the three groups. Specifically, T2DM samples exhibited a reduced proportion of beta cells and a trend toward increased alpha cells compared to ND controls, consistent with known islet dysfunction in diabetes.

### 3.2 Screening key diabetes-related genes using LASSO regression

**FIGURE 2:**
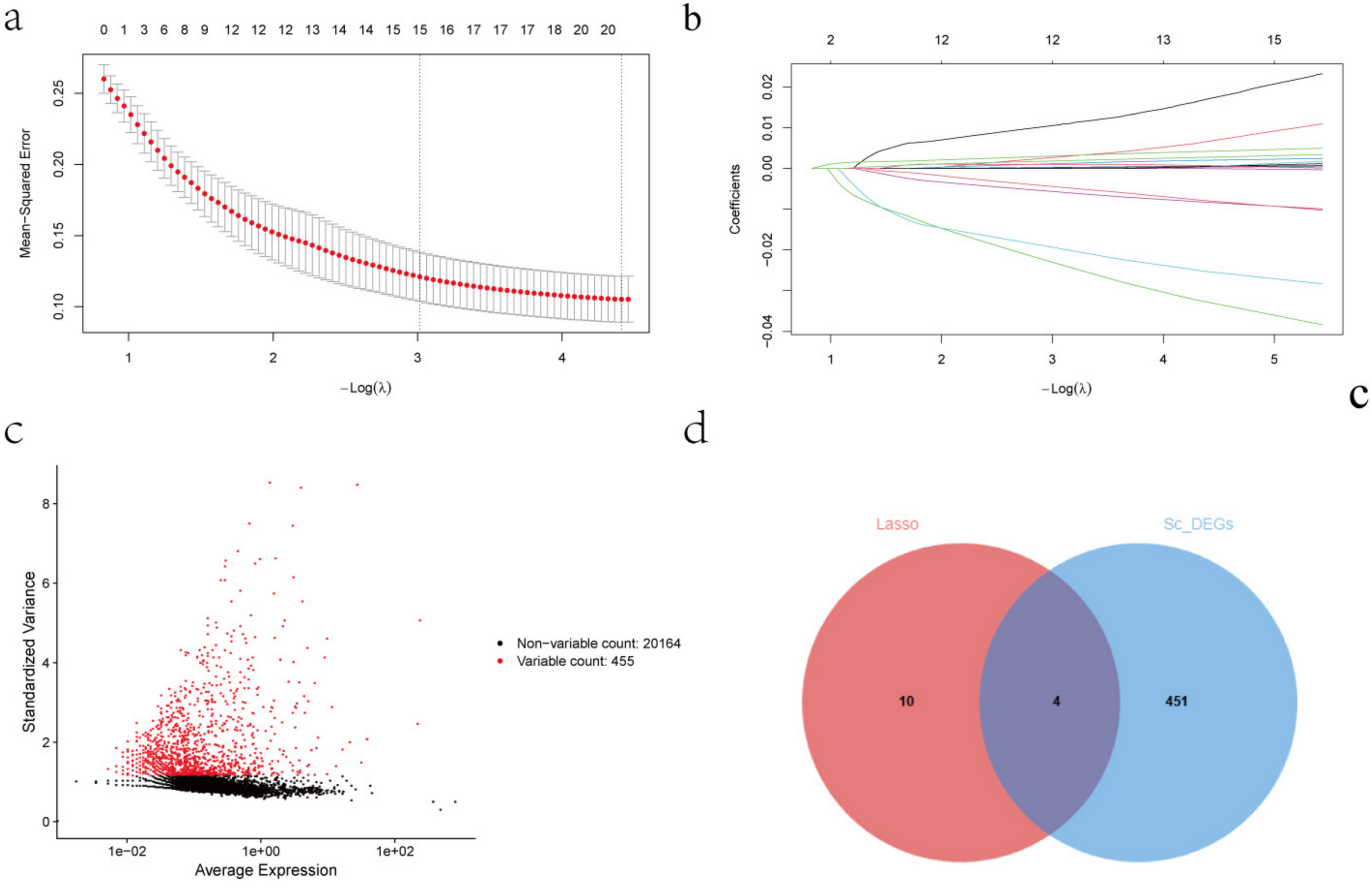
Feature gene screening results based on the LASSO regression model and differential gene analysis. **(a)** Five-fold cross-validation curve showing the model’s mean squared error corresponding to different λ values; the dashed line marks the optimal λ. **(b)** LASSO regression coefficient path plot, showing the changes in regression coefficients for each gene as the penalty coefficient λ varies. **(c)** Volcano plot of DEGs, showing the distribution of significantly up/down-regulated genes among the 455 highly variable genes. **(d)** Venn diagram showing the intersection between the 455 DEGs and the 14 genes screened by LASSO, identifying four common genes: PNLIP, BUB1, CTSB, NAMPT.

To identify core genes highly associated with diabetes, a LASSO regression model was employed for feature selection. As shown in Figure 2a, the optimal regularization parameter lambda (λ) was determined via 5-fold cross-validation. This value minimized the model’s Mean Squared Error, effectively controlling model complexity and reducing the risk of overfitting while maintaining predictive performance. In the coefficient path plot (Figure 2b), it can be observed that as the λ value increased, the regression coefficients of most genes were compressed to zero, while only a few key genes retained non-zero coefficients, highlighting the superiority of LASSO in feature selection.

We further integrated the results of highly variable gene analysis and identified 455 DEGs. A volcano plot illustrates the overall trend of gene expression changes (Figure 2c). Venn diagram analysis revealed an overlap of four genes between the 455 DEGs and the 14 key genes selected by LASSO regression: PNLIP, BUB1, CTSB, NAMPT (Figure 2d). These four genes may play critical roles in the development of T2DM, and their regression coefficients indicate their relative importance in different pathological processes. These results provide a reliable basis for screening diabetes-related biomarkers and indicate directions for subsequent mechanistic studies.

### 3.3 ROC curve validation of the diagnostic efficacy of key genes

**FIGURE 3:**
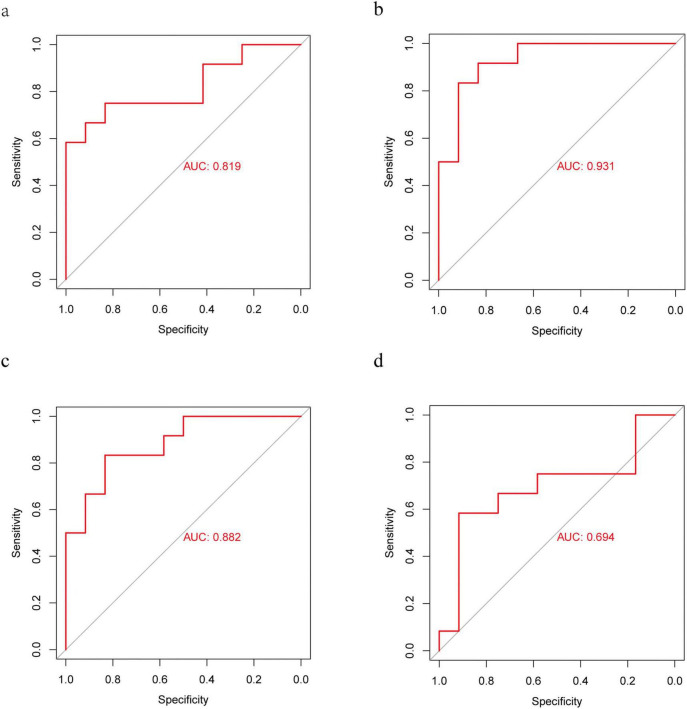
ROC analysis validation of key genes in the GSE29221 dataset. **(a)** PNLIP, AUC = 0.819; **(b)** BUB1, AUC = 0.931; **(c)** CTSB, AUC = 0.882; **(d)** NAMPT, AUC = 0.694. All curves indicate potential diagnostic value in distinguishing the two sample types.

To evaluate the potential diagnostic value of the aforementioned four key genes for T2DM, Receiver Operating Characteristic (ROC) curve analysis was performed using the independent dataset GSE29221. The results showed that the Area Under the Curve (AUC) for the BUB1 gene reached 0.931 (Figure 3b), indicating extremely high diagnostic accuracy with excellent sensitivity and specificity. The AUC for the CTSB gene was 0.882 (Figure 3c), also demonstrating excellent discriminatory ability. The AUC value for the PNLIP gene was 0.819 (Figure 3a), showing good diagnostic potential. Although NAMPT had a relatively lower AUC (0.694, Figure 3d), it still possessed some discriminatory ability between the diabetic and normal groups. Overall, the four genes exhibited a gradient of diagnostic efficacy, providing a theoretical basis for developing multi-gene combination biomarkers for clinical translation.

### 3.4 KEGG and GO enrichment analysis reveals functional mechanisms

**FIGURE 4:**
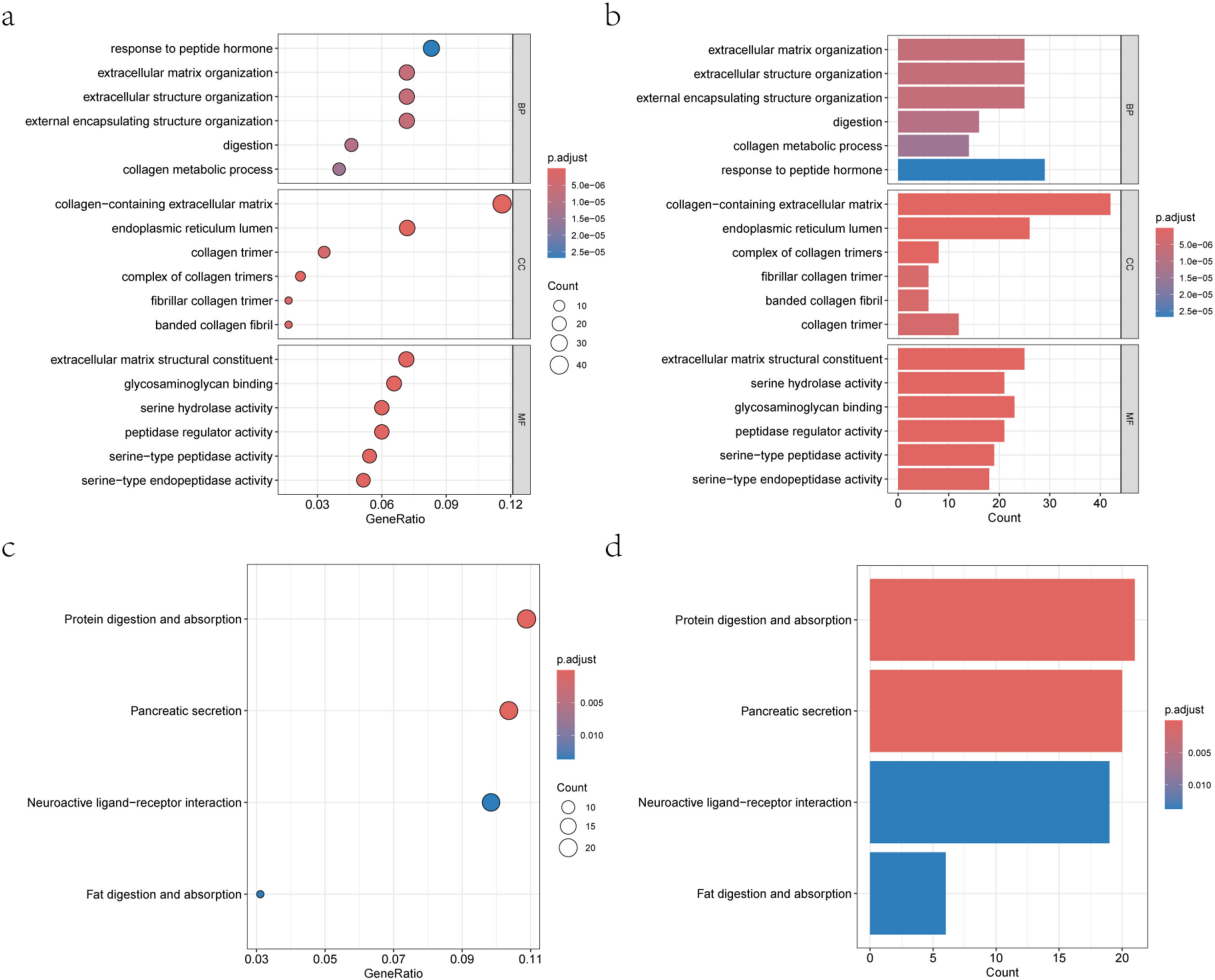
KEGG and GO functional enrichment analysis results. **(a)** Bubble plot of GO enrichment analysis, displaying significantly enriched Biological Process, Molecular Function, and Cellular Component terms. **(b)** Bar plot of GO enrichment, showing the gene count and enrichment significance for representative terms. **(c)** Bubble plot of KEGG pathway enrichment, highlighting metabolic and signaling pathways related to T2DM. **(d)** Bar plot of KEGG enrichment, showing the enrichment status of key pathways such as fat digestion and absorption, and pancreatic secretion.

To further explore the biological functions of the screened genes and the involved pathological mechanisms, GO and KEGG enrichment analyses were performed. The GO analysis covered three aspects: BP, CC, and MF. The results showed that in Biological Processes, the relevant genes were significantly enriched in collagen metabolism, digestive processes, and extracellular matrix organization, among others (Figures 4a,b). Regarding Molecular Function, they were primarily involved in serine-type endopeptidase activity and peptidase regulator activity. Cellular Component analysis indicated that these genes were mainly located in the endoplasmic reticulum lumen and the collagen-containing extracellular matrix.

KEGG pathway analysis further identified multiple metabolism and signaling pathways closely related to T2DM, including fat digestion and absorption, neuroactive ligand-receptor interaction, pancreatic secretion, and protein digestion and absorption (Figures 4c,d). These pathways not only reflect key aspects of material metabolism and energy balance but also suggest the important role of immune regulation and inflammatory responses in T2DM development. The comprehensive enrichment analysis results indicate that the identified feature genes participate in the occurrence and development of T2DM at multiple levels, including extracellular matrix remodeling, nutrient absorption, and cellular signal transduction, providing a systematic functional background and theoretical support for subsequent mechanistic studies.

### 3.5 Cell communication network analysis reveals intercellular interactions

**FIGURE 5:**
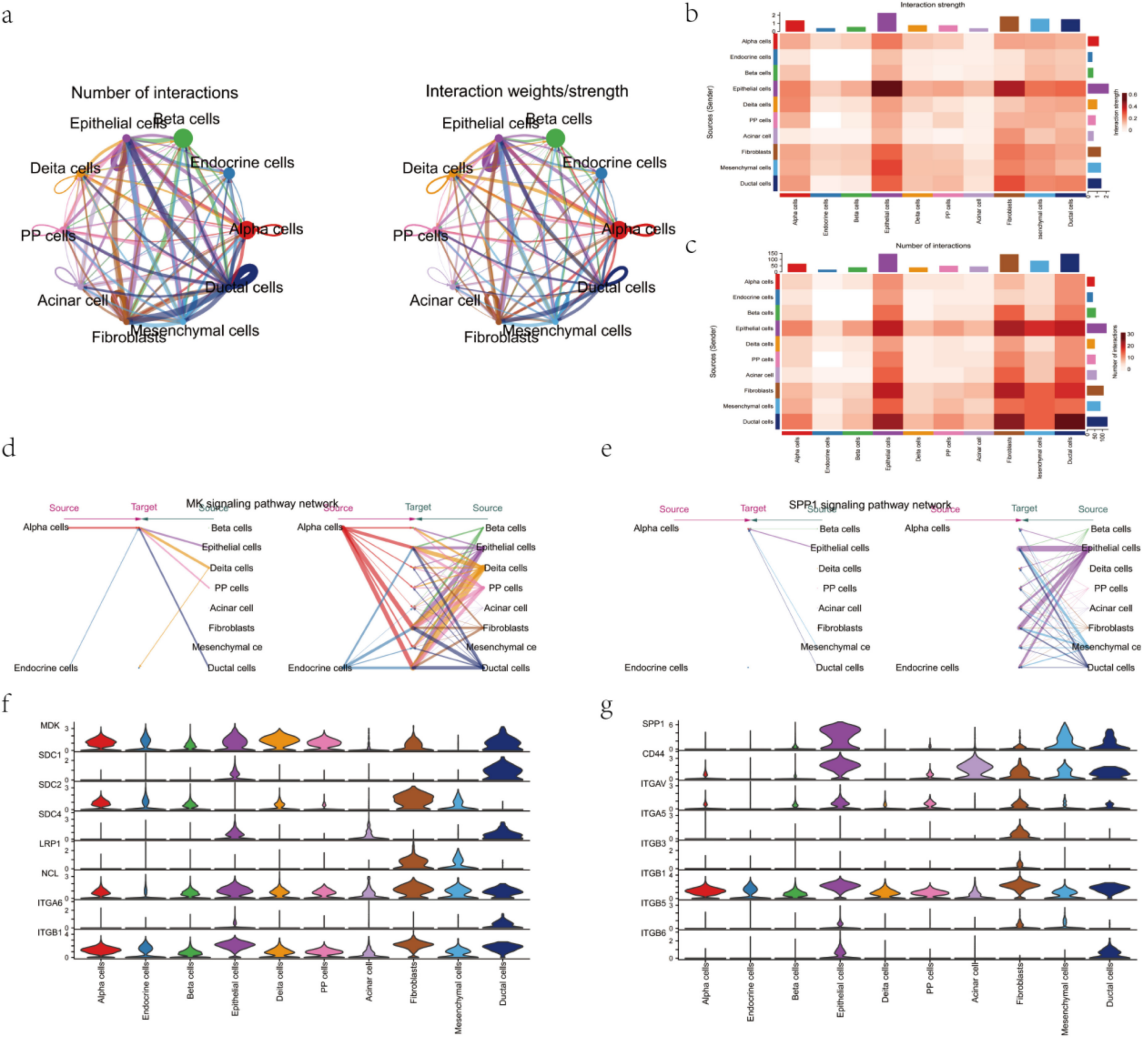
Cell-cell communication network analysis. **(a)** Network diagram showing the number and strength of interactions between different cell types. **(b,c)** Heatmaps of cell communication network strength and number of interactions, displaying the communication status between each cell pair. **(d)** Specific expression pattern of the MK pathway across cell types. **(e)** Specific expression pattern of the SPP1 pathway across cell types. **(f)** Expression levels of the MK pathway in various cell types. **(g)** Expression levels of the SPP1 pathway in various cell types.

To gain a deeper understanding of the information exchange mechanisms between cells in the diabetic microenvironment, we systematically analyzed the intercellular communication network using tools like CellChat. The results showed significant heterogeneity in the number and strength of interactions between different cell types (Figures 5a–c). The communication strength between certain cell pairs (e.g., Alpha–Beta cells) was significantly higher than other combinations, forming interaction “hotspots” suggesting their potential core role in maintaining islet function and regulating insulin secretion.

Focusing further on key signaling pathways, the MK and SPP1 pathways showed distinct cell type-specific expression patterns. The MK pathway promotes cell proliferation and differentiation by activating downstream signals, while the SPP1 pathway is primarily involved in extracellular matrix remodeling and cell migration processes (Figures 5d–g). Functionally, these two pathways complement each other, jointly regulating T2DM-related microenvironment changes. Network topology analysis indicated that Alpha and Beta cells act as signaling hubs, coordinating the behavior of surrounding cells through multi-directional signal flows, reinforcing their central role in maintaining islet homeostasis. This part of the research reveals the dynamic changes in the intercellular communication network during the pathogenesis of T2DM from a systems level, providing new perspectives for targeted interventions aimed at cell-cell interactions. Additionally, CTSB is associated with the SPP1 signaling pathway, as SPP1 can upregulate CTSB expression. In diabetic kidney disease (DKD), NAMPT can form a receptor-ligand pair and participate in the regulation of multiple signaling pathways.

### 3.6 Experimental validation of key gene expression in clinical samples

**FIGURE 6:**
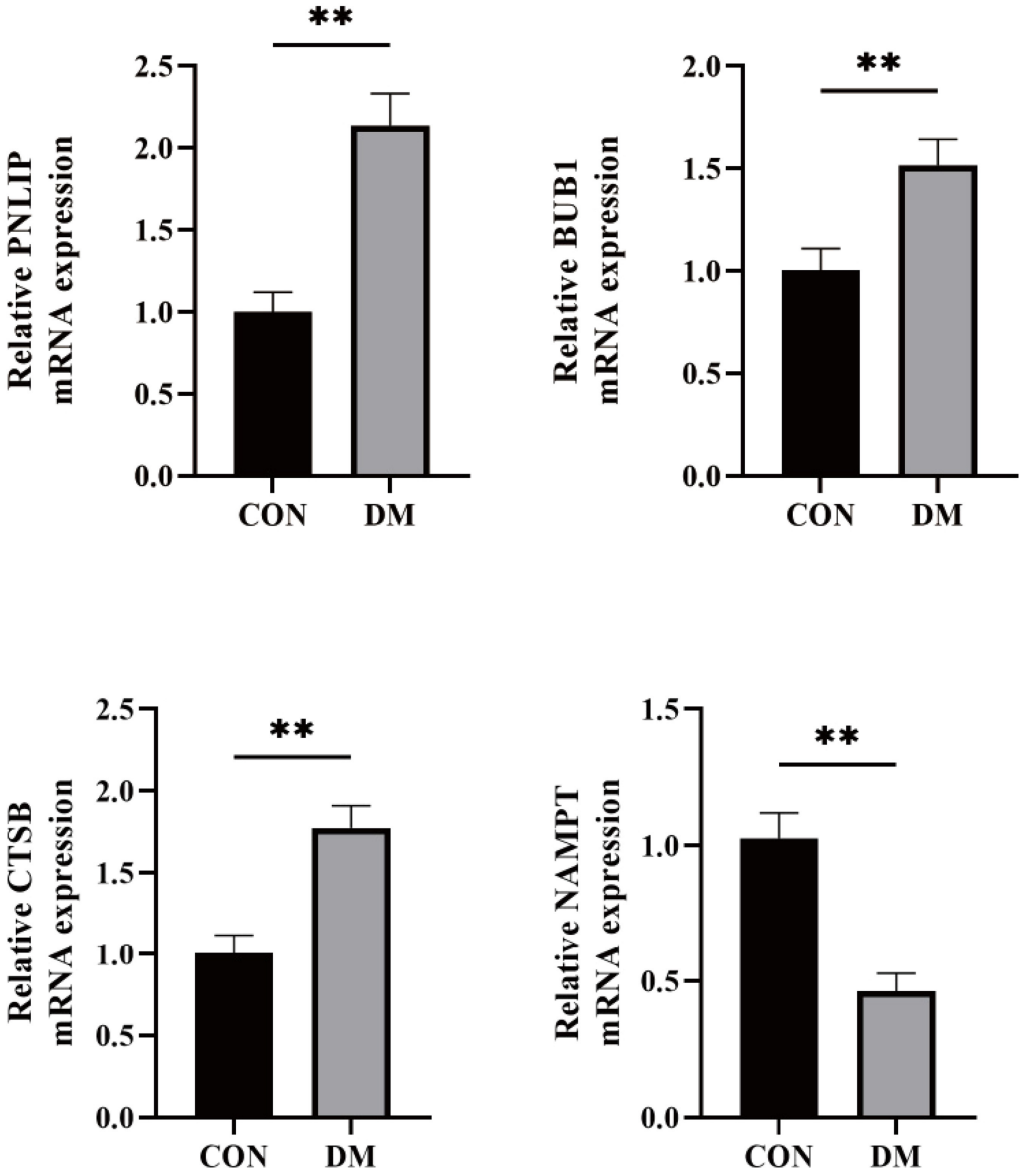
qRT-PCR to verify gene expression in serum. qRT-PCR detection of PNLIP, BUB1, CTSB, and NAMPT expression levels in peripheral blood samples from 15 diabetic patients and 20 healthy controls. **p < 0.01.

To validate the reliability of the bioinformatics analysis results, and considering that peripheral blood is an easily accessible sample type in clinical practice, we aimed to explore whether these signature genes identified in the pancreatic context also carry detectable diagnostic signals in the circulatory system. Although PNLIP has traditionally been regarded as a pancreas-specific enzyme, studies have indicated that it can leak into the blood under specific pathological conditions and subsequently be detected in the bloodstream. Similarly, BUB1, CTSB, and NAMPT are widely expressed genes involved in cell cycle regulation, lysosomal function, and NAD + biosynthesis, respectively, and documented evidence exists regarding their expression in extra-pancreatic tissues such as hematopoietic and circulating cells. We collected peripheral blood samples from 15 diabetic patients and 20 healthy controls, isolated serum, and extracted total RNA from the samples. The mRNA expression levels of the four genes PNLIP, BUB1, CTSB, and NAMPT were detected using qRT-PCR. The results showed that compared with the healthy control group, the expressions of PNLIP, BUB1, and CTSB were significantly up-regulated, while the expression of NAMPT was significantly down-regulated in diabetic patients (Figure 6, *p* < 0.01). This experiment confirms the aberrant expression of these genes in the diabetic state at the clinical sample level, further supporting their potential as biomarkers.

## 4 Discussion

Diabetes, as a complex metabolic disease, has long had its comprehensive molecular characterization hindered by the cellular heterogeneity of tissue samples. The maturation of scRNA-seq technology offers a novel perspective to address this challenge, enabling the revelation of disease-specific gene expression patterns at single-cell resolution (3). By integrating scRNA-seq data with machine learning algorithms, this study identified four core candidate genes-PNLIP, BUB1, CTSB, and NAMPT-and evaluated their diagnostic potential. While the results suggest a promising diagnostic model, the findings should be interpreted as hypothesis-generating, and they contribute to a deeper, albeit preliminary, understanding of diabetic pathological mechanisms at the cellular level.

Single-cell transcriptome analysis is a key technique for deciphering cellular heterogeneity within the pancreatic islet microenvironment in diabetes. This study identified 10 cell types, consistent with previous research; for example, clustering tools like Seurat can accurately quantify the loss of pancreatic β-cells and immune cell infiltration in T2DM (23, 24). This approach effectively overcomes the inherent limitation of traditional bulk sequencing, which “averages” signals from different cell types, thereby providing a reliable cell-specific context for subsequent analysis. Furthermore, machine learning algorithms (such as Random Forest) demonstrate strong capability in handling such high-dimensional data and optimizing cell classification (3, 17). The preliminary analysis in this study laid the foundation for this, ensuring that the signature gene screening was based on an accurate cell atlas.

In the phase of signature gene screening and validation, this study employed a strategy combining differential expression analysis with LASSO regression, refining four core genes from 455 candidates. LASSO regression, utilizing L1 regularization to penalize coefficients, effectively handles high-dimensional, collinear gene data, thereby selecting the most informative feature combination. This method’s reliability has been widely validated in the discovery of diabetes biomarkers (25, 26). Notably, similar integrative approaches combining differential expression analysis with machine learning have identified shared key genes between T2DM and comorbid conditions, supporting the robustness of our methodology (6, 8, 9). ROC analysis on an independent dataset suggested promising but variable diagnostic performance (AUCs: 0.694-0.931). Notably, the AUC of BUB1 (0.931) and CTSB (0.882) exceeded that of HbA1c (∼0.85), the current clinical standard for diabetes diagnosis, suggesting potential complementary value. However, given the small validation cohort (*n* = 6), these results should be interpreted cautiously. Furthermore, the variable diagnostic efficacy across the four genes (AUC range: 0.694–0.931) hints at potential utility in diabetes subtyping, as distinct gene expression patterns may reflect different pathophysiological endotypes. This marks a solid step from computational prediction toward clinical application.

Functional enrichment analysis provided clear biological significance for these four core genes. They were significantly enriched in pathways such as extracellular matrix (ECM) remodeling, digestion and absorption, and signal transduction. Numerous studies indicate that ECM remodeling is a core pathological process in pancreatic islet fibrosis and various diabetic complications (e.g., nephropathy), while aberrant signaling pathways directly participate in insulin resistance and β-cell dysfunction (25, 27). For instance, some studies using scRNA-seq have found that specific genes (e.g., ESR1) drive T2DM progression by affecting insulin signaling pathways (24). Additionally, cell communication analysis in this study revealed the central roles of Alpha and Beta cells within the interaction network. This aligns with findings from studies using tools like CellPhoneDB, suggesting that aberrant communication between β-cells and immune cells is a key factor disrupting islet functional homeostasis (28). This aligns with emerging evidence that shared molecular signatures and signaling pathways underlie T2DM and its associated malignancies, where common therapeutic targets may be exploited (7, 8). These analyses place the isolated gene markers within an organic biological network, elucidating the potential mechanisms of their synergistic action.

The final experimental validation serves as a bridge connecting computational discoveries to clinical translation. This study confirmed through qRT-PCR that the expression levels of the four core genes (PNLIP, BUB1, CTSB, and NAMPT) were altered in the peripheral blood of diabetic patients. This design is clinically prospective, as peripheral blood, being an easily accessible liquid biopsy source, is ideal for achieving non-invasive diagnosis. This result is highly consistent with the emphasis placed on wet-lab validation within the field (20, 29). Many studies follow a similar path: initial bioinformatic prediction followed by confirmation of gene expression changes in clinical samples using techniques like qRT-PCR or Western blot to assess their stability as biomarkers (20, 30). The upregulated pattern of these genes in this study and their association with immune cell infiltration further suggest their potential active involvement in regulating the immune microenvironment of diabetes (31, 32).

Specifically, missense variants in PNLIP promote chronic pancreatitis (CP) by increasing protease sensitivity, and during pancreatic injury, PNLIP leakage into adipose tissue hydrolyzes triglycerides, generating excess free fatty acids leading to adipocyte necrosis and potentially exacerbating common diabetic comorbidities (e.g., heart failure), thus primarily acting as an indirect risk factor for T3c diabetes (33–36). BUB1 might not directly participate in glucose metabolism but could influence the development of diabetes-related cancer complications by mediating immunosuppressive pathways (37, 38). NAMPT plays a complex “dual role” in T2DM. Intracellularly, it serves as the rate-limiting enzyme for NAD + synthesis; its downregulation correlates with insulin resistance, whereas activation restores NAD + levels and improves metabolic homeostasis (39–41). Extracellularly, secreted eNAMPT (particularly the monomeric form) functions as a pro-inflammatory cytokine that impairs pancreatic β-cell function and exacerbates insulin resistance (42, 43). This paradoxical nature renders NAMPT an attractive therapeutic target: enhancing its enzymatic activity may ameliorate metabolism, while suppressing its inflammatory form could protect islet function (41–44). Its role in gestational diabetes remains significant, though underlying mechanisms require further elucidation (45). Additionally, in a mouse model of diabetic cardiomyopathy (DCM), cardiac CTSB expression was significantly elevated. Gene knockout of CTSB resulted in improved cardiac function, suggesting that CTSB may exacerbate myocardial injury by mediating apoptosis or inflammatory responses. However, its specific role in the context of T2DM still requires further investigation (46). The identification of such pleiotropic genes resonates with studies revealing shared genetic architecture between T2DM and multiple comorbid diseases, suggesting potential for broader therapeutic applications (6, 9).

In summary, this study integrated scRNA-seq with machine learning approaches to systematically identify and preliminarily validate potential key signature genes associated with T2DM. However, several limitations should be acknowledged. First, although the signature genes were derived from pancreatic islet scRNA-seq data, their validation used peripheral blood samples. The biological relevance and translational mechanisms between these compartments remain unclear. Second, the independent ROC validation dataset (GSE29221) includes only three T2DM patients and three controls, limiting statistical power and generalizability. A potential concern is validating islet-derived genes using skeletal muscle data. Nevertheless, this cross-tissue validation yielded robust AUCs, especially for BUB1, suggesting systemic T2DM processes rather than islet-specific changes. Muscle is a key insulin resistance site with shared pathways; thus, the signature may serve as a cross-compartment biomarker. Future studies should validate in pancreatic-specific cohorts and explore BUB1’s function in both tissues. Third, the clinical validation cohort, though showing significant differences, is modest (15 T2DM, 20 controls) and single-center. Fourth, this study is primarily computational and descriptive; the causal roles of these four genes in diabetes pathogenesis have not been experimentally tested. Larger multi-center cohorts are needed to validate the diagnostic model, and cellular/animal models are required to dissect their functional roles in cell-cell interactions and establish their potential as liquid biopsy markers for early diagnosis or diabetes subtyping.

## 5 Conclusion

Leveraging scRNA-seq and machine learning, this study systematically identified and preliminarily validated a set of potential key signature genes for T2DM. By resolving cellular heterogeneity, we identified 10 cell types in the T2DM microenvironment. Using LASSO regression, we refined four core genes-PNLIP, BUB1, CTSB, and NAMPT-from 455 DEGs. ROC analysis on an independent dataset suggested promising but variable diagnostic performance (AUCs: 0.694–0.931). Functional enrichment analysis indicated their potential involvement in processes such as extracellular matrix remodeling, while cell communication analysis suggested the central role of Alpha and Beta cells within the signaling network. qRT-PCR validation confirmed significantly up-regulated expression of PNLIP, BUB1, CTSB, and down-regulated NAMPT in T2DM patients. These findings provide new, albeit preliminary, evidence for understanding diabetic mechanisms and developing candidate biomarkers, warranting further validation in larger cohorts and functional studies.

## Data Availability

The original contributions presented in this study are included in the article/Supplementary material, further inquiries can be directed to the corresponding author.

## Footnotes

1
https://www.ncbi.nlm.nih.gov/geo/

2
https://ggplot2.tidyverse.org/

3
https://cran.r-project.org/package=glmnet

4
https://cellchat.org/

5
https://cran.r-project.org/package=pROC
