# Supplementary material for: Identification and preliminary clinical validation of type 2 diabetes signature genes through machine learning analysis of scRNA-seq data
Source: Front Med (Lausanne). 2026 May 12;13:1801737. doi: 10.3389/fmed.2026.1801737 (PMC13201128; doi:10.3389/fmed.2026.1801737)
Supplement: Supplementary file 1 [file Table_1.docx]

library(dplyr)

library(Seurat)#单细胞数据分析

library(patchwork)#将多个ggplot图像组合成一个图

library(ggplot2)

library(SingleR)#对单细胞数据进行自动注释

library(CCA)

library(clustree)

library(cowplot)

library(monocle)

library(tidyverse)

library(SCpubr)

library(GSEABase)

library(harmony)

library(plyr)

getwd()

setwd('D:/益居康项目/YJK20251030G6-深圳糖尿病-唐芳')

################读取并构建seurat对象################

{

data_dir <- paste0(getwd(),"/data") # 定义数据存储的路径

samples = list.files(data_dir) # 列出数据文件夹中的样本文件

dir = file.path(data_dir, samples) # 构造文件路径

afdata <- Read10X(data.dir = dir)

#创建seurat对象

yjsl <- CreateSeuratObject(counts = afdata,

project = "SeuratObject",

min.cells = 3,#基因需要在至少3个细胞中表达

min.features = 200)#细胞中至少要有200个基因被检测到

#将细胞身份信息赋值给Type列，便于后续处理

afidens=mapvalues(Idents(yjsl), from = levels(Idents(yjsl)), to = samples)

Idents(yjsl)=afidens

yjsl$Type=Idents(yjsl)

}

samples

# 根据Type组的样本名字定义新的group组

yjsl$group <- ifelse(yjsl$Type %in% c("GSM6846489", "GSM6846494","GSM6846495"), "NC",

ifelse(yjsl$Type %in% c("GSM6846488", "GSM6846490","GSM6846491"), "DIA", NA))

# 查看前几行数据，确认分组信息

head(yjsl@meta.data)

{

################质控可视化################

#计算线粒体基因的百分比

yjsl[["percent.mt"]] <- PercentageFeatureSet(yjsl, pattern = "^MT-")

#计算核糖体基因的百分比

yjsl[["percent.rb"]] <- PercentageFeatureSet(yjsl, pattern = "^RP")

#绘制小提琴图，展示每个细胞中的基因数（nFeature_RNA）、检测到的分子数（nCount_RNA）以及线粒体基因的百分比（percent.mt）

VlnPlot(yjsl, features = c("nFeature_RNA", "nCount_RNA","percent.mt"),

ncol = 3)#,pt.size = 0

##nCount表示每个细胞中检测到的分子数

##nFeature表示每个细胞中检测到的基因数

###上述两者，若有异常的离群细胞则说明该细胞可能为双细胞或多细胞（即，质控目的为剔除异常值）

## 绘制散点图，比较不同特征之间的关系

plot2 <- FeatureScatter(yjsl, feature1 = "nCount_RNA", feature2 = "percent.rb")+ RotatedAxis()

plot2

plot3 <- FeatureScatter(yjsl, feature1 = "nCount_RNA", feature2 = "nFeature_RNA")+ RotatedAxis()

plot3

plot2 + plot3

###过滤基因

sub1 <- yjsl$nCount_RNA >= 1000 ## 过滤掉低于1000的细胞

sub2 <- yjsl$nFeature_RNA >= 200 & yjsl$nFeature_RNA <= 10000# 过滤掉基因数小于200或大于10000的细胞

sub3 <- yjsl$percent.mt <= 20# 过滤掉线粒体基因百分比大于20%的细胞

sub4<-yjsl$percent.rb<= 20 # 过滤掉核糖体基因百分比大于20%的细胞

sub <-sub2 & sub3 & sub4 # 合并过滤条件

yjsl <- yjsl[, sub]# 应用过滤条件，保留符合条件的细胞

}

#重新绘制

VlnPlot(yjsl, features = c("nFeature_RNA", "nCount_RNA","percent.mt"),

ncol = 3)

#######使用LogNormalize方法#######

yjsl <- NormalizeData(yjsl, normalization.method = "LogNormalize",

scale.factor = 10000)

#######鉴定高变基因#######

yjsl <- FindVariableFeatures(yjsl, selection.method = "vst", nfeatures = 2000)

#提取前15的高变基因

top15 <- head(VariableFeatures(yjsl), 15)

top15

##展示高变基因

plot1 <- VariableFeaturePlot(yjsl, pt.size = 2, raster = T)

plot1

plot2 <- LabelPoints(plot = plot1, points = top15,

xnudge = 0,

ynudge = 0)

plot2

######数据Scaling，降维前的必要准备

{

all.genes <- rownames(yjsl)

yjsl <- ScaleData(yjsl, features = all.genes)

}

####正式降维###

yjsl<- RunPCA(yjsl, features = VariableFeatures(object =yjsl))

#数据可视化方法

#VizDimReduction

VizDimLoadings(yjsl, dims = 1:2, reduction = "pca")

#DimHeatmap

DimHeatmap(yjsl, dims = 1:15, cells = 500, balanced = TRUE)

#ElbowPlot

ElbowPlot(yjsl, ndims=20, reduction="pca")

########harmony去除批次效应########################

yjsl <- RunHarmony(yjsl, group.by.vars = "Type")

########下面的步骤都是一样的########

#######鉴定高变基因#######

yjsl <- FindVariableFeatures(yjsl, selection.method = "vst", nfeatures = 2000)

#提取前15的高变基因

top15 <- head(VariableFeatures(yjsl), 15)

top15

##展示高变基因

plot1 <- VariableFeaturePlot(yjsl, pt.size = 2, raster = T)

plot1

plot2 <- LabelPoints(plot = plot1, points = top15,

xnudge = 0,

ynudge = 0)

plot2

######数据Scaling，降维前的必要准备

all.genes <- rownames(yjsl)

yjsl <- ScaleData(yjsl, features = all.genes)

####正式降维###

yjsl<- RunPCA(yjsl, features = VariableFeatures(object =yjsl))

#数据可视化方法

#DimHeatmap

DimHeatmap(yjsl, dims = 1:15, cells = 500, balanced = TRUE)

#ElbowPlot（拐点可确定维度）

ElbowPlot(yjsl, ndims=20, reduction="pca")

########确定维度、细胞分群########

#选择PC

PC=1:15

###根据上一步中的合适维度对细胞进行分群

yjsl <- FindNeighbors(yjsl, dims = PC)

#resolution可以设0.1-1之间，值越高，亚群数目越多，常规0.5

yjsl <- FindClusters(yjsl, resolution =seq(0.2,1.2,0.1))

clustree(yjsl)

##!!!记住这个维度

yjsl <- FindClusters(yjsl, resolution =0.5)

###使用UMAP及进行非线性降维

yjsl <- RunUMAP(yjsl, dims = PC)

#数据可视化

DimPlot(yjsl, reduction = "umap", label = T,repel = T)

DimPlot(yjsl, reduction = "umap", label = T,repel = T,split.by = 'group')

###使用tSNE进行非线性降维

yjsl = RunTSNE(yjsl, dims = PC)

embed_tsne <- Embeddings(yjsl, 'tsne')

DimPlot(yjsl, reduction = "tsne")

DimPlot(yjsl, reduction = "tsne",split.by = 'group')

###进行正常组和肿瘤组的差异分析

# 将group列赋值为Seurat对象的身份

##记住，后面分析要经常运行这行代码

Idents(yjsl) <- yjsl$group

# 进行差异基因分析

{

DEGs <- FindMarkers(yjsl, ident.1 = 'DIA', ident.2 = 'NC', logfc.threshold = 1)#可调节

}

# 将分组信息添加到差异基因表格

DEGs$group <- ifelse(DEGs$avg_log2FC > 0, "DIA", "NC") # 根据log2FoldChange决定分组

# 输出差异基因结果

write.csv(DEGs, "DIA和正常组差异基因.csv")

## 过滤p值和调整后的p值

DEGs.filter <- subset(DEGs, p_val_adj < 0.05)

write.csv(DEGs.filter, "过滤后Tumor_vs_NC_差异基因.csv")

##重新赋予回来

Idents(yjsl) <- yjsl$seurat_clusters

###大体看一下情况###

# 查看多少个类型

table(yjsl@meta.data$seurat_clusters)

#只保留上调差异表达的基因

markers <- FindAllMarkers(yjsl, only.pos = TRUE,

min.pct = 0.25,

logfc.threshold = 0.25)

#markers_Type <- FindAllMarkers(yjsl, only.pos = F, min.pct = 0.25, logfc.threshold = 0.25)

save(yjsl,file = "yjsl_Type.rda")

write.csv(markers,file = "cluster的标记基因.csv")

head(markers)

##保存用于后续分析

saveRDS(yjsl,"af2.rds")

library(randomcoloR)

##专门美化图片的包

#install.packages("/Users/zhangzhenhu/Desktop/单细胞注释_人工/scRNAtoolVis_0.1.0.tar.gz", repos=NULL, type="source")

# devtools::install_github('junjunlab/scRNAtoolVis')

library(scRNAtoolVis)

# yjsl=readRDS("af2.rds")

################推荐：人工手动注释################

######第一种，通过差异基因################

# 找到所有差异表达基因

Idents(yjsl) <- yjsl$seurat_clusters

allmarkers <- FindAllMarkers(yjsl, only.pos = TRUE,

min.pct = 0.25, #基因在至少 25% 的细胞中表达才会被考虑为差异基因

logfc.threshold = 0.25)#只选择 log2 fold change 大于 0.25 的基因

save(allmarkers,file = "allmarkers.rda")

# load("allmarkers.rda")

# 提取每个簇中具有最显著差异表达的前4个基因

top4_markers <- allmarkers %>%

group_by(cluster) %>%

top_n(n = 4, wt = avg_log2FC)

#换个颜色的热图

DoHeatmap(yjsl, features = unique(top4_markers$gene)) + NoLegend()+

scale_fill_gradientn(colors = c("#2fa1dd", "white", "#f87669"))

#气泡图

DotPlot(yjsl, features = unique(top4_markers$gene),cols = "RdYlBu") +

RotatedAxis()

VlnPlot(yjsl, features = "CD3E",group.by="cellType")+NoLegend()

## umap单个基因图

FeaturePlot(yjsl,features = "CD3E")

###然后可以进一些注释网站

#CellMarker: http://xteam.xbio.top/CellMarker/

######第二种常见的标记基因################

# T Cells (CD3D, CD3E, CD8A)

#

# B cells (CD19, CD79A, MS4A1 [CD20])

#

# Plasma cells (IGHG1, MZB1, SDC1, CD79A)

#

# Monocytes and macrophages (CD68, CD163, CD14)

#

# NK Cells (FGFBP2, FCG3RA, CX3CR1)

#

# Photoreceptor cells (RCVRN)

#

# Fibroblasts (FGF7, MME)

#

# Endothelial cells (PECAM1, VWF)

#

# epi or tumor (EPCAM, KRT19, PROM1, ALDH1A1, CD24)

#

# immune (CD45+,PTPRC)

#

# epithelial/cancer (EpCAM+,EPCAM)

#

# stromal (CD10+,MME,fibo or CD31+,PECAM1,endo)

#

# mast cells( TPSAB1 and TPSB2 )

#

# naive B cells(MS4A1 (CD20), CD19, CD22, TCL1A, and CD83)

#

# plasma B cells(CD38, TNFRSF17 (BCMA), and IGHG1/IGHG4

#######看别人的文献################

yjsl <- FindClusters(yjsl, resolution = 0.5)##记住和上面resolution数目一样

# Pancreas cell type marker genes

genes <- list(

"Beta cell" = c("INS", "IAPP", "PCSK1", "PAX6", "NKX6-1"),

"Alpha cell" = c("GCG", "IRX2", "MAFB", "ARX", "TTR"),

"Delta cell" = c("SST", "HHEX", "RBP4", "PAX6"),

"PP cell" = c("PPY", "FOXA1", "PAX6"),

"Acinar cell" = c("PRSS1", "CPA1", "CTRB1", "CELA3A", "AMY2A"),

"Ductal cell" = c("KRT19", "CFTR", "MUC1", "SOX9", "SPP1"),

"Endocrine cell" = c("CHGA", "CHGB", "PCSK1", "SCG5"),

"Epithelial cell" = c("EPCAM", "KRT8", "KRT18", "KRT19"),

"Fibroblast" = c("COL1A1", "COL1A2", "DCN", "LUM", "ACTA2"),

"Mesenchymal cell" = c("VIM", "FN1", "COL3A1", "S100A4"),

"Progenitor cell" = c("SOX9", "PDX1", "HES1", "NGN3"),

"Stem cell" = c("NANOG", "POU5F1", "SOX2", "KLF4"),

"Cancer stem cell" = c("CD44", "ALDH1A1", "PROM1", "SOX9"),

"Neuroendocrine cell" = c("CHGA", "SYP", "ENO2", "SCG2")

)

#genes <- list("T cells" = c("CD3D","CD2"),

# "Macrophages"= c("CD163", "CD14"),

# "B cells" = c("CD79A", "MS4A1"),

# "Fibroblasts" = c("ACTA2", "PDGFRB"),

# "Endothelial cells" = c("VWF","RAMP2"),

# "Mast cells"=c("TPSAB1","TPSB2"),

# "Epithelial cells"=c("SFTPA1", "EPCAM")

#

#)

do_DotPlot(sample = yjsl,features = genes,dot.scale = 10,legend.length = 50,

legend.framewidth = 2, font.size =10)

#另一种颜色气泡图

DotPlot(yjsl, features = genes,cols = "RdYlBu") +

RotatedAxis()

#人工注释

table(yjsl@active.ident)

ann.ids <- c("Alpha cells", #cluster0

"Alpha cells", #cluster1

"Endocrine cells", #以下按顺序操作

"Beta cells",

"Beta cells",

"Epithelial cells",

"Deita cells",

"Beta cells",

"Beta cells",

"PP cells",

"Endocrine cells",

"Alpha cells",

"Beta cells",

"Acinar cell",

"Fibroblasts",

"Mesenchymal cells",

"Endocrine cells",

"Beta cells",

"Ductal cells"

)

afidens=mapvalues(Idents(yjsl), from = levels(Idents(yjsl)), to = ann.ids)

Idents(yjsl)=afidens

yjsl$cellType=Idents(yjsl)

#########人工注释后结果可视化

# 可视化UMAP/tSNE

DimPlot(yjsl, reduction = "umap", label = T, label.size = 3.5)+theme_classic()+theme(panel.border = element_rect(fill=NA,color="black", size=0.5, linetype="solid"),

legend.position = "right")

DimPlot(yjsl, reduction = "tsne", label = T, label.size = 3.5)+theme_classic()+theme(panel.border = element_rect(fill=NA,color="black", size=0.5, linetype="solid"),legend.position = "right")

DimPlot(yjsl, reduction = "tsne",split.by = 'group')

###############################细胞通讯

library(Seurat)

library(tidyverse)

library(Matrix)

library(stringr)

library(dplyr)

library(Seurat)

library(patchwork)

library(ggplot2)

library(SingleR)

library(CCA)

library(clustree)

library(cowplot)

library(monocle)

library(tidyverse)

library(SCpubr)

library(harmony)

library(plyr)

library(randomcoloR)

library(CellChat)

load("yjsl_Type.rda")

head(yjsl@meta.data)

#创建cellchat对象

cellchat = createCellChat(object = yjsl,

group.by = "cellType")#通过 group.by 定义分组

#展示以下现在的细胞分组

levels(cellchat@idents)

# #细胞亚群各组数量

# group <- as.numeric(table(cellchat@idents))

#设置配体受体交互数据库

CellChatDB <- CellChatDB.human #如果是老鼠的话使用内置“CellChatDB.mouse”数据

showDatabaseCategory(CellChatDB)

#人的数据包括61.8%的旁分泌/自分泌信号互作、

#21.7%的细胞外基质(ECM)受体互作

#16.5%的细胞-细胞通讯互作

unique(CellChatDB$interaction$annotation)

#如果想用全部的用于cellchat分析，不进行subsetDB，直接指定cellchat@DB <- CellChatDB 即可。

# CellChatDB.use <- subsetDB(CellChatDB, search = "Secreted Signaling")

# # set the used database in the object

# cellchat@DB <- CellChatDB.use

# use Secreted Signaling for cell-cell communication analysis

CellChatDB.use <- subsetDB(CellChatDB, search = "Secreted Signaling")

cellchat@DB <- CellChatDB.use

######################对表达数据进行预处理######################

##This step is necessary even if using the whole database

cellchat <- subsetData(cellchat)

#根据配置设置

future::plan("multisession", workers = 4)

options(future.globals.maxSize = 4 * 1024^3) # 4GB

{

# 识别过表达基因(很慢)

cellchat <- identifyOverExpressedGenes(cellchat)

# 识别过表达配体受体对

cellchat <- identifyOverExpressedInteractions(cellchat)

}

{

# 将配体、受体投射到PPI网络（很慢）

#projectData函数将配体受体对的表达值投射到PPI上为可选项，做了该步骤的话可以在data.project中查看结果

data(PPI.human)

library(Matrix)

PPI.human <- as(PPI.human, "sparseMatrix")

cellchat <- smoothData(cellchat, adj = PPI.human)

#cellchat <- projectData(cellchat, PPI.human)

##########数据和配体受体库准备好之后，就可以根据表达值推断细胞类型之间的互作##########

## 推断细胞通讯网络（极慢，5分钟）

cellchat <- computeCommunProb(cellchat, raw.use = TRUE)#raw.use = TRUE 表示使用raw数据，而不使用上一步projectData后的结果

##官网代码（population.size = TRUE时候。CellChat可以在概率计算中考虑每个细胞群中细胞比例的影响）

#cellchat <- computeCommunProb(cellchat, raw.use = TRUE, population.size = TRUE)

save.image()

###如果特定细胞群中只有少数细胞，则过滤掉细胞间的通信（极慢，5分钟）

cellchat <- filterCommunication(cellchat, min.cells = 5)

}

#建议保存一下

save(cellchat,file = "cellchat.rda")

load("cellchat.rda")

##提取 保存结果

df.net <- subsetCommunication(cellchat)

head(df.net)

write.csv(df.net,"df.net.csv")

#df.net1 <- subsetCommunication(cellchat,slot.name = "netP")

##查看细胞通讯分群###

levels(cellchat@idents)

########3种方式建立网络########

##通过序号

df.net1 <- subsetCommunication(cellchat, sources.use = c(1,2), targets.use = c(4,5))

head(df.net1)

##通过名字

df.net2 <- subsetCommunication(cellchat, sources.use = c("T cells"), targets.use = c("Mast cells" ,"Mast cells"))

head(df.net2)

##通过通路

df.net3 <- subsetCommunication(cellchat, signaling = c("EGF"))

head(df.net3)

############计算cell-cell communication#####

#使用computeCommunProbPathway计算每个信号通路的所有配体-受体相互作用的通信结果，结存存放在net 和 netP中

cellchat <- computeCommunProbPathway(cellchat)

##使用aggregateNet计算细胞类型间整合的细胞通讯结果

cellchat <- aggregateNet(cellchat)

##########快乐的可视化过程##########

##########1、celltype之间通讯结果##########

##########通讯次数（左）通讯强度(右)

groupSize <- as.numeric(table(cellchat@idents))

par(mfrow = c(1,2), xpd=TRUE)

netVisual_circle(cellchat@net$count, vertex.weight = groupSize,

weight.scale = T,

label.edge= F, title.name = "Number of interactions")

netVisual_circle(cellchat@net$weight, vertex.weight = groupSize,

weight.scale = T,

label.edge= F, title.name = "Interaction weights/strength")

#左图：外周各种颜色圆圈的大小表示细胞的数量，圈越大，细胞数越多。

#发出箭头的细胞表达配体，箭头指向的细胞表达受体。配体-受体对越多，线越粗。

#右图：互作的概率或者强度值（强度就是概率值相加）

##换一种可视化方式

p3 <- netVisual_heatmap(cellchat)

p3

p4 <- netVisual_heatmap(cellchat, measure = "weight")

p4

p3 + p4

##########分别展示

#根据celltype的个数，灵活调整mfrow = c(2,3) 参数

mat <- cellchat@net$weight

###另一种

{

mat <- cellchat@net$count

par(mfrow = c(2,5), xpd=TRUE)

for (i in 1:nrow(mat)) {

mat2 <- matrix(0, nrow = nrow(mat), ncol = ncol(mat), dimnames = dimnames(mat))

mat2[i, ] <- mat[i, ]

netVisual_circle(mat2, vertex.weight = groupSize, weight.scale = T, edge.weight.max = max(mat), title.name = rownames(mat)[i])

}

}

##########单个信号通路可视化

#展示当前有哪些通路结果

cellchat@netP$pathways

#选择感兴趣的进行展示

pathways.show <- c("TGFb")

#查看当前的celltype顺序

levels(cellchat@idents)

#通过vertex.receiver指定target 的细胞类型

##左边是你选定的细胞群，右边是剩下的细胞群

vertex.receiver = c(1,2)

##层次图

netVisual_aggregate(cellchat, signaling = "TGFb",

vertex.receiver = vertex.receiver,layout="hierarchy")

#在层次图中，实体圆和空心圆分别表示源和目标。圆的大小与每个细胞组的细胞数成比例。线越粗，互作信号越强。

##圈图

par(mfrow=c(1,1))

netVisual_aggregate(cellchat, signaling ="TGFb", layout = "circle")

##和弦图

par(mfrow=c(1,1))

netVisual_aggregate(cellchat, signaling ="TGFb", layout = "chord", vertex.size = groupSize)

##热图

par(mfrow=c(1,1))

netVisual_heatmap(cellchat, signaling = "TGFb", color.heatmap = "Reds")

##########气泡图##############################

#通过sources.use 和 targets.use指定定受体-配体

levels(cellchat@idents)

netVisual_bubble(cellchat, sources.use = 5,

targets.use = c(1,2,3), remove.isolate = FALSE)

##指定受体-配体细胞类型且指定通路

cellchat@netP$pathways

netVisual_bubble(cellchat, sources.use = c(3,5), targets.use = c(1,2,4,6),

signaling = c("TGFb","SPP1"), remove.isolate = FALSE)

#某条信号通路（如SPP1）的所有基因在细胞群中的表达情况展示

plotGeneExpression(cellchat, signaling = "SPP1")
